# Minimally-Invasive Versus Abdominal Hysterectomy for Endometrial Carcinoma With Glandular or Stromal Invasion of Cervix

**DOI:** 10.3389/fonc.2021.670214

**Published:** 2021-05-20

**Authors:** Jihee Jung, Joseph J. Noh, Chel Hun Choi, Tae-Joong Kim, Jeong-Won Lee, Byoung-Gie Kim, Duk-Soo Bae, Yoo-Young Lee

**Affiliations:** Gynecologic Cancer Center, Department of Obstetrics and Gynecology, Samsung Medical Center, Sungkyunkwan University School of Medicine, Seoul, South Korea

**Keywords:** endometrial cancer, minimally-invasive surgery, laparotomy, disease-free survival, overall survival

## Abstract

The purpose of the study was to evaluate the feasibility of laparoscopic approach versus laparotomy in endometrial cancer that extends to the cervix in the form of glandular extension and/or stromal invasion. A retrospective, single-center cohort study was conducted using data between 1995 and 2017 at an urban tertiary academic medical center. We identified patients who were diagnosed with endometrial cancer whose tumor involved the uterine cervix on final pathology. Operative and oncologic outcomes were compared between the patients who underwent minimally-invasive surgery (MIS) versus those who underwent laparotomy. A total of 282 patients with endometrial cancer were reviewed for the study. Among these patients, 76 patients underwent hysterectomy and surgical staging *via* MIS. There was no conversion from MIS to laparotomy. In the MIS group, shorter hospital stay (4.4 ± 2.3 days for MIS group *vs*. 7.1 ± 4.7 days for laparotomy group; *p*-value = 0.002) and less blood loss during the operations (228 mL *vs*. 478 mL, *p*-value < 0.001) were observed compared to the laparotomy group. The multivariate Cox regression analysis revealed that age at diagnosis, FIGO stage, histology grades, tumor size, lymph-vascular space invasion were independent prognostic markers for poor oncologic outcomes but the types of surgical approach (MIS *vs*. laparotomy) were not associated with it. The means by which colpotomy was performed (either intracorporeal or transvaginal) among the MIS group also did not affect patient survivals. Among the women with endometrial cancer that involved the uterine cervix, surgical treatment *via* MIS compared to laparotomy showed no difference in survival outcomes but better perioperative results. These findings support the use of MIS for these patient group.

## Introduction

Endometrial cancer is the sixth most common malignancy worldwide and the most common gynecological malignancy in developed countries with new 380,000 patients diagnosed worldwide in 2018 (1). The incidence of endometrial cancer is increasing due to increasing rates of obesity and life expectancy. Risk factors of endometrial cancer include the use of hormone therapy, diabetes, having fewer children and history of breast cancer (2, 3). In almost 80% of women, the disease is detected in the early stages, which results in cure rates greater than 90% (4).

The current standard treatment of endometrial cancer is total hysterectomy and bilateral salpingo-oophorectomy. The staging procedure encompasses pelvic and para-aortic lymph node assessment (either by dissection or sentinel lymph node mapping if feasible), omentectomy and peritoneal biopsy, depending on histologic type and stage. Traditionally, laparotomy was used for surgical treatment, but since the 2000s, the frequency of performing laparoscopic approach has increased. Many studies have demonstrated the safety and feasibility of laparoscopic approach in early stages of endometrial cancer (5). For example, studies reported that laparoscopic hysterectomy was associated with less wound infection and blood loss, shorter hospital stay compared to laparotomy and demonstrated no significant difference in overall survival (OS) and disease-free survival (DFS) (4, 6, 7). Most studies, however, were limited to patients with early FIGO (International Federation of Obstetrics and Gynecology) stages (6, 7). Therefore, the recommendation of laparoscopic approach for endometrial cancer surgery in professional guidelines is limited for those with early stages of the disease (8).

The results of the Laparoscopic Approach to Cervical Cancer (LACC) trial, a phase III multi-center randomized trial, were reported in 2018, surprisingly showing inferior survivals of minimally-invasive surgery (MIS) for early cervical cancer compared to laparotomy (9). One potential explanation to understand the inferior oncologic outcomes of MIS in early cervical cancer is related to the surgical techniques of MIS such as frequent manipulations of tumor on the cervix with uterine elevator and intra-abdominal colpotomy which might allow tumor spillage into the abdominal cavity during the procedure.

Endometrial cancer can extend to the cervix in the form of glandular extension and/or stromal invasion. Tumor extension of endometrial cancer to the cervix may also generate the same concerns for inferiority of MIS seen in the LACC trial. Unfortunately, there is a lack of studies investigating the safety of MIS in this subset of patients. In the present study, we retrospectively reviewed endometrial cancer patients who had cervical invasion on final pathology and compared the oncologic outcomes between the two surgical approaches. We also performed analysis to investigate whether either intracorporeal or transvaginal colpotomy was associated with poor survival outcomes.

## Materials and Methods

The study was approved by the Institutional Review Board (IRB number 2020-10-131-001). This was a retrospective cohort study including patients with endometrial cancer who were histologically confirmed with cervical stromal invasion and/or glandular extension on final pathology. They all underwent staging operations between January 1995 and December 2017 at an urban academic tertiary medical center in Seoul, South Korea (Samsung Medical Center).

Women with biopsy-confirmed or clinically suspicious endometrial cancer underwent either laparotomic or laparoscopic hysterectomy for staging. Radical hysterectomy could be performed if cervical stromal invasion was highly suspicious on computed tomography (CT), magnetic resonance imaging (MRI) or by physical examination. From 2006, laparoscopic staging was introduced in the present institution, and in 2009, more than half of endometrial cancer surgeries were done with laparoscopy. However, the decision on the type of hysterectomy (Type I *vs*. II *vs*. III) and the route of hysterectomy (MIS *vs*. laparotomy) was decided at the surgeons’ discretion. If there was a conversion from MIS to laparotomy, we considered it a case of laparotomy. MIS included laparoscopy-assisted vaginal hysterectomy (LAVH), laparoscopy-assisted radical vaginal hysterectomy (LARVH), total laparoscopic hysterectomy (TLH), and laparoscopic radical hysterectomy (LRH). Robot surgery was also considered as MIS.

The demographic parameters evaluated were age at diagnosis, body mass index (BMI), and parity. Information about the types of surgery, conversion rates, the duration of surgery (from skin incision to skin closure), estimated blood loss, hemoglobin levels, postoperative hospitalization days, postoperative pain levels expressed by numeric rating scale (NRS), intra- and postoperative complications, and the types of adjuvant therapy were obtained. Clinical and pathological variables were stages (2018 FIGO stages), grade, histopathologic type, depth of myometrial invasion (as < 50% or ≥ 50%), lymph node involvement, lymph-vascular space invasion (LVSI), number of lymph nodes yielded, and survival outcomes. OS and DFS were also assessed. DFS was defined as the time between the first treatment and recurrence, death or last follow-up, whichever occurred first. OS was defined as the time interval from the day of surgery to the date of death or last follow-up.

The *Shapiro-Wilk test* was used to test normality of the data. Mean ± standard deviation was used for normal distributions and median (range) was used for non-normal distributions. Frequency distributions among categorical variables were compared using the *Chi-Squared test* or *Fisher’s exact test*. Survival curves were calculated according to the *Kaplan-Meier methods* with the *log-rank test*. The *Cox proportional hazards model* was used for multivariate analysis to assess different prognostic factors. A *p*-value less than 0.05 was considered statistically significant. Statistical analysis was performed with SPSS software (Version 21.0; SPSS Inc., Chicago, IL, USA).

## Results

**Figure 1:**
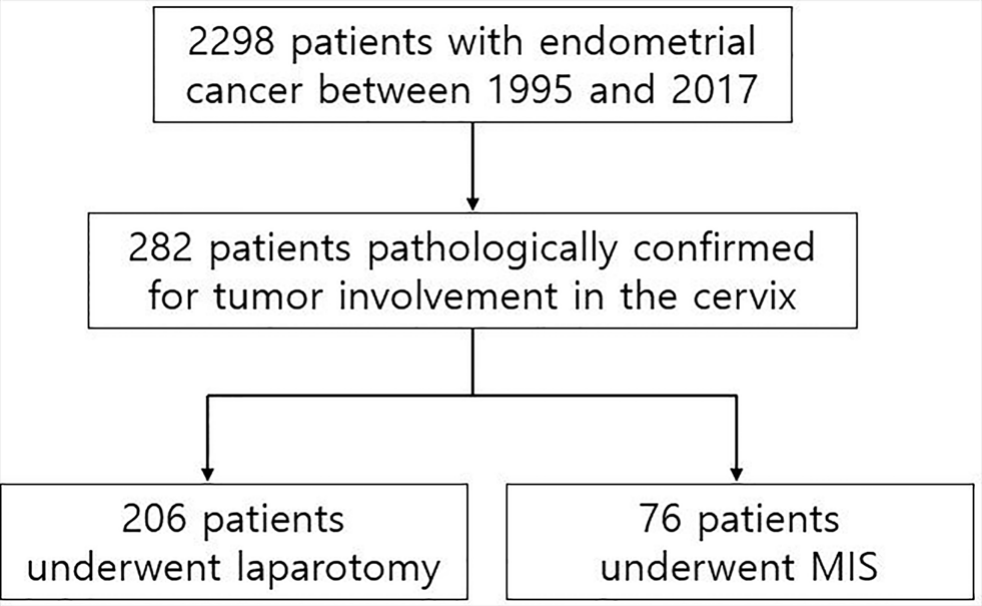
Flowchart of the patient selection.

**Figure 2:**
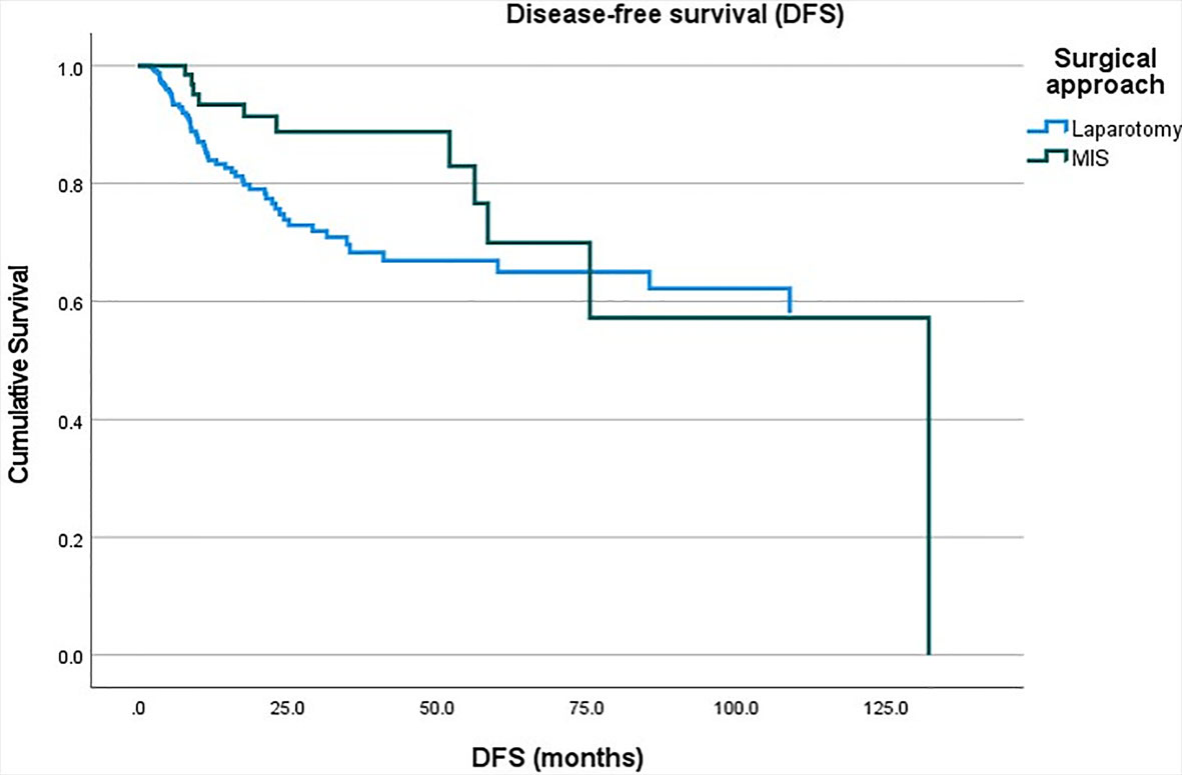
Disease-free survival of the patients with endometrial cancer between the minimally-invasive surgery (MIS) group *vs*. laparotomy group.

**Figure 3:**
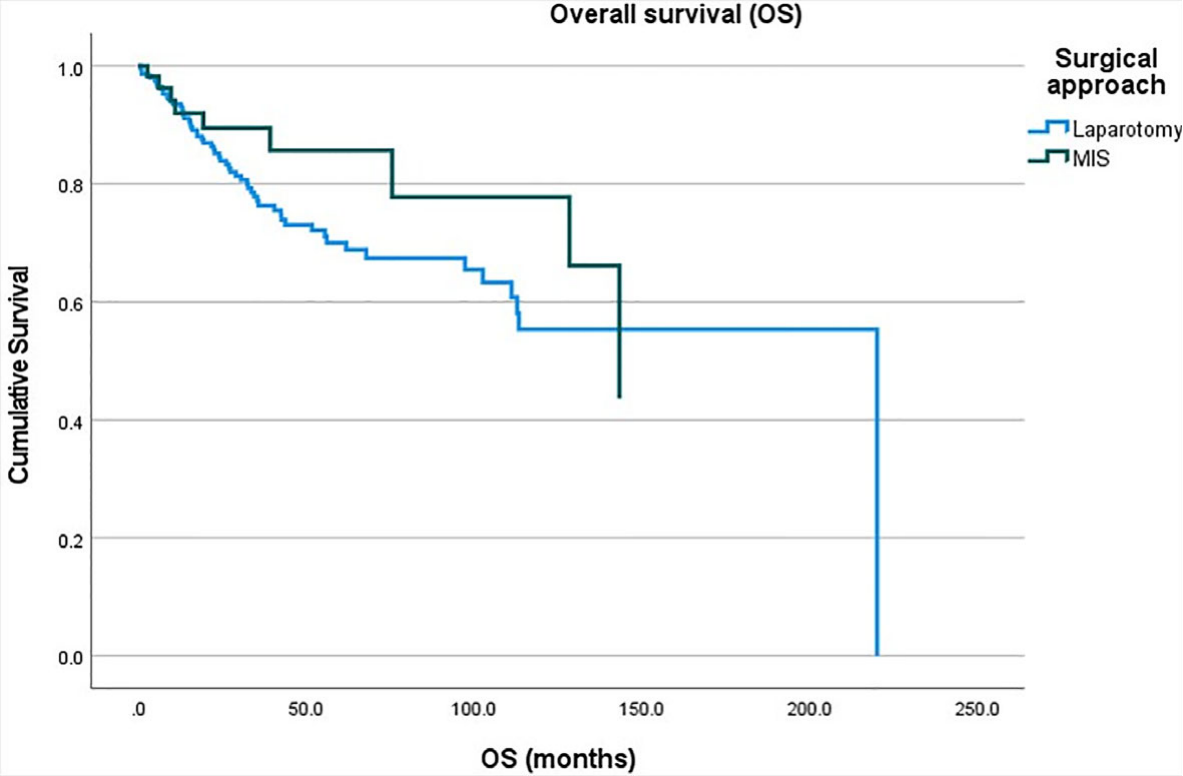
Overall survival of the patients with endometrial cancer between the minimally-invasive surgery (MIS) group *vs*. laparotomy group.

**Figure 4:**
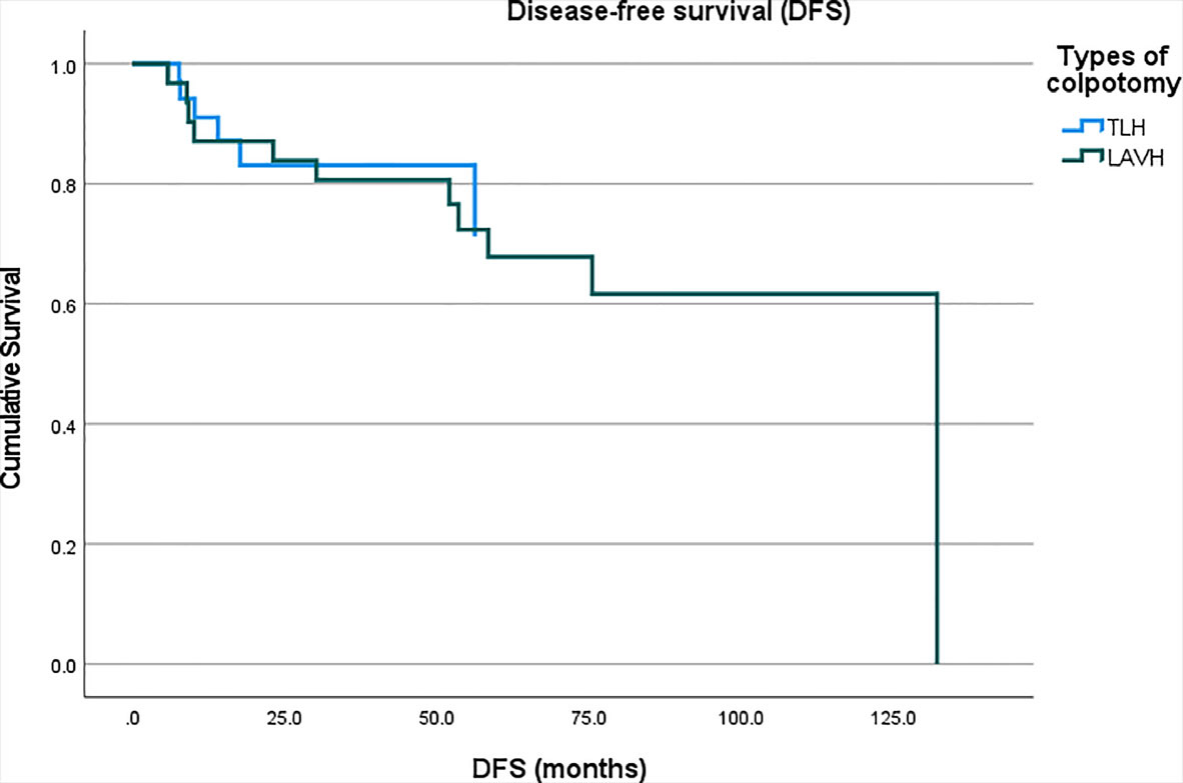
Comparison of disease-free survival of the patients with endometrial cancer who underwent intracorporeal colpotomy (TLH, total laparoscopic hysterectomy) *vs*. transvaginal colpotomy (LAVH, laparoscopy-assisted vaginal hysterectomy).

**Figure 5:**
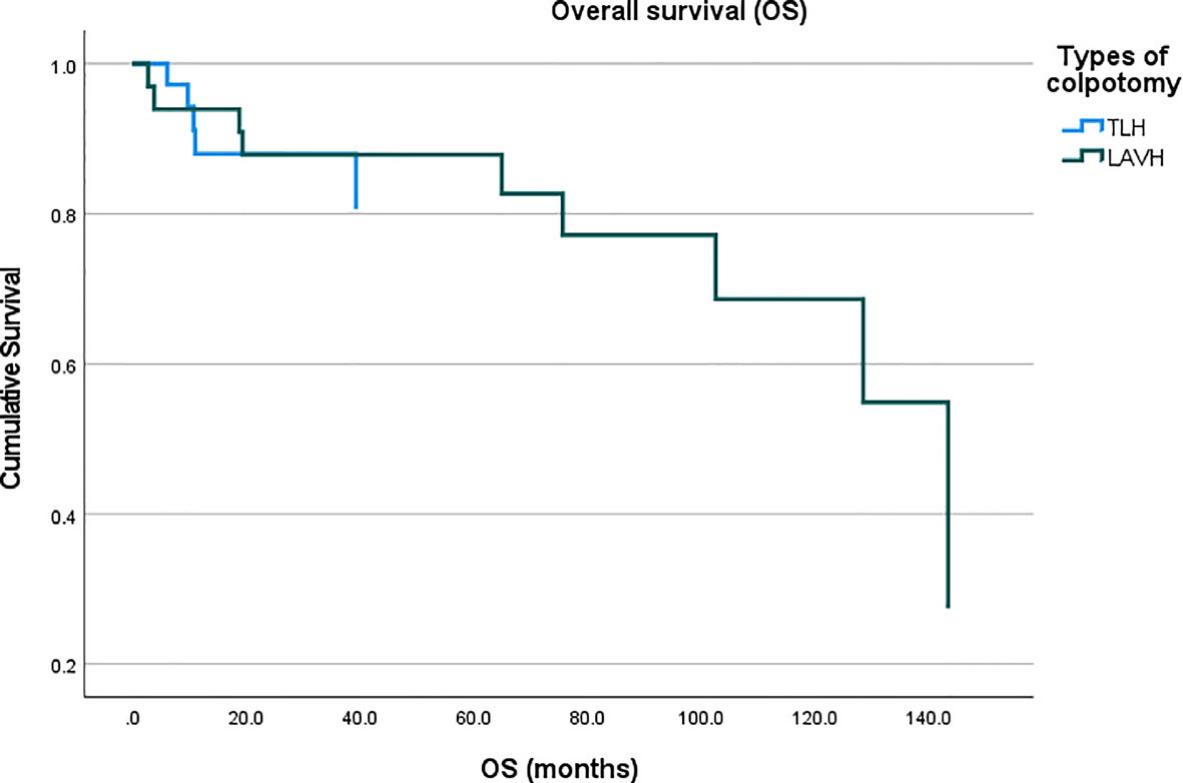
Comparison of overall survival of the patients with endometrial cancer who underwent intracorporeal colpotomy (TLH, total laparoscopic hysterectomy) *vs*. transvaginal colpotomy (LAVH, laparoscopy-assisted vaginal hysterectomy).

A total of 2,298 patients were identified who had completed surgical staging for endometrial cancer during the study period. Among them, 282 patients (12.3%, 282/2,298) were confirmed with tumor invasion to the uterine cervix. Of the 282 patients, 76 patients underwent MIS (27.0%, 76/282) while 206 patients underwent laparotomy (73.0%, 206/282) for staging (**Figure 1**). In the MIS group, LAVH was the most common surgical approach (52%) followed by LRH (24%), robotic hysterectomy (12%), LARVH (8%), and TLH (4%). **Table 1** shows the baseline characteristics of the patients (**Table 1**). Compared to the patients in the MIS group, those in the laparotomy group were older (55.4 ± 11.5 *vs*. 51.3 ± 10.7, *p*-value: 0.007), had lighter body weight (58.1 ± 9.3 *vs*. 61.3 ± 12.3, *p*-value: 0.022), and had higher CA-125 levels (153.5 ± 414.8 *vs*. 13.4 ± 14.9, *p*-value: 0.005). The difference in the CA-125 levels between the two groups was presumably due to more advanced stages of the disease in the laparotomy group. Pathologic findings after the surgeries were compared between the two groups (**Table 2**). As it was reflected by the higher tumor marker levels of the laparotomy group in pre-operative evaluations, it was found that the disease status of the patients in the laparotomy group was more advanced than that of the patients in the MIS group. More patients in the laparotomy group had advanced FIGO stages, higher histology grades, deeper depth of myometrial invasion, adnexal metastasis, intraperitoneal tumor metastasis, and larger tumor size. Although differences in cellular differentiation grades were observed between the two groups as seen in **Table 2**, no statistical differences were shown in terms of histology types. The Kaplan-Meier survival analysis revealed no statistical differences in DFS or OS between the two groups (**Figures 2** and **3**). The Cox proportional hazards model revealed that age at diagnosis, FIGO stage, histology grade, tumor size, and LVSI were independent prognostic markers for poor DFS while age at diagnosis, FIGO stage, histology grade, and LVSI were prognostic markers for poor OS (**Tables 3** and **4**). Types of surgical approach (MIS *vs.* laparotomy) or methods of colpotomy (intracorporeal *vs*. transvaginal) did not affect DFS or OS (**Figures 4** and **5**). We also performed subgroup analysis with those patients who were found to be FIGO stage II on their final pathology excluding the patients with other disease stages. The Cox proportional hazards models with the same variables were performed, which revealed similar results as the analysis that included all stage patients (**Supplementary Tables 1** and **2**).

**Table 1:** Baseline characteristics of the patients.

	Laparotomy (N=206)	MIS^†^ (N=76)	Total (N=282)	*p*-value
**Age at diagnosis (years)**	55.4 ± 11.5	51.3 ± 10.7	54.3 ± 11.5	0.007
**Body weight (kg)**	58.1 ± 9.3	61.3 ± 12.3	59.0 ± 10.3	0.022
**Height (cm)**	156.6 ± 6.3	156.1 ± 6.7	156.5 ± 6.4	0.506
**BMI^†^ (kg/m_2_)**	23.7 ± 3.6	25.2 ± 5.0	24.1 ± 4.0	0.005
**Concurrent cancer**				
* Ovarian cancer*	3 (1.46%)	0	3 (1.06%)	0.706
* Colorectal cancer*	4 (1.94%)	2 (2.63%)	6 (2.13%)	
* Other gynecologic cancers*	2 (0.97%)	0	2 (0.71%)	
* Breast cancer*	6 (2.91%)	3 (3.95%)	9 (3.19%)	
* None*	187 (90.78%)	69 (90.79%)	256 (90.78%)	
**Menopause at diagnosis**				
* No*	92 (44.66%)	41 (53.95%)	133 (47.16%)	0.166
* Yes*	114 (55.34%)	35 (46.05%)	149 (52.84%)	
**Hormone replacement therapy**				
* Never*	201 (97.57%)	76 (100%)	277 (98.22%)	0.598
* Past user*	3 (1.45%)	0	3 (1.06%)	
* Current user*	1 (0.48%)	0	1 (0.35%)	
**Tamoxifen use**				
* No*	205 (99.51%)	75 (98.68%)	280 (99.29%)	0.461
* Yes*	1 (0.48%)	1 (1.31%)	2 (0.70%)	
**Diabetes mellitus**				
* No*	176 (62.41%)	70 (92.10%)	246 (87.23%)	0.137
* Yes*	30 (10.63%)	6 (7.89%)	36 (12.76%)	
**Hypertension**				
* No*	153 (74.27%)	62 (81.57%)	215 (76.24%)	0.201
* Yes*	53 (25.72)	14 (18.42%)	67 (23.75%)	
**Dyslipidemia**				
* No*	204 (99.02%)	71 (93.42%)	275 (97.51%)	0.007
* Yes*	2 (0.97%)	5 (6.57%)	7 (2.48%)	
**Endometrial hyperplasia**				
* No*	201 (97.57%)	73 (96.05%)	274 (97.16%)	0.716
* Simple without atypia*	1 (0.48%)	0	1 (0.35%)	
* Complex without atypia*	1 (0.48%)	1 (1.31%)	2 (0.70%)	
* Simple with atypia*	0	0	0	
* Complex with atypia*	3 (1.45%)	2 (2.63%)	5 (1.77%)	
**Pre-operative CA-125 (U/mL)**	153.5 ± 414.8	13.4 ± 14.9	115.8 ± 359.9	0.005
**Pre-operative CA 19-9 (U/mL)**	150.4 ± 501.3	33.8 ± 65.8	124.3 ± 444.6	0.344
**Pre-operative CEA^†^ (ng/mL)**	7.4 ± 29.6	7.3 ± 5.2	6.8 ± 5.4	0.212
**Cervical involvement of cancer on pre-operative imaging**	86 (41.7%)	25 (32.5%)	111 (39.2%)	0.155

^†^MIS, minimally-invasive surgery; BMI, body mass index; CEA, carcinoembryonic antigen.

**Table 2:** Pathologic findings and adjuvant treatments.

	Laparotomy (N=206)	MIS^†^ (N=76)	Total (N=282)	*p*-value
**Treatment type**				
* Surgery*	25 (12.13%)	10 (13.15%)	35 (12.41%)	0.810
* Surgery + RT* ^†^	85 (41.26%)	33 (43.42%)	118 (41.84%)	
* Surgery + CCRT* ^†^	39 (18.93%)	16 (20.05%)	55 (19.50%)	
* Surgery + CT* ^†^	56 (27.19%)	16 (20.05%)	72 (25.53%)	
**Types of hysterectomy**				
* Type I*	120 (58.25%)	55 (72.36%)	175 (62.05%)	0.072
* Type II*	33 (16.01%)	6 (7.89%)	39 (13.82%)	
* Type III*	53 (25.72%)	15 (19.73%)	68 (24.16%)	
**FIGO^†^ stage**				
* Stage I*	30 (14.56%)	19 (25.00%)	49 (17.37%)	0.011
* Stage II*	70 (33.98%)	23 (30.26%)	93 (32.97%)	
* Stage III*	62 (30.09%)	25 (32.89%)	87 (30.85%)	
* Stage IV*	37 (17.96%)	3 (3.94%)	40 (14.18%)	
* No data*	5 (2.42%)	4 (5.26%)	9 (3.19%)	
**Histology**				
* Endometrioid*	126 (61.16%)	58 (76.31%)	184 (65.24%)	0.355
* Papillary serous*	18 (8.73%)	7 (9.21%)	25 (8.86%)	
* Mucinous*	1 (0.48%)	1 (1.31%)	2 (0.70%)	
* Clear cell*	6 (2.91%)	0	6 (2.12%)	
* Squamous cell*	1 (0.48%)	0	1 (0.35%)	
* MMMT* ***^†^***	24 (11.65%)	4 (5.26%)	28(9.92%)	
* Undifferentiated*	4 (1.94%)	0	4 (1.41%)	
* High-grade EST* ***^†^***	1 (0.48%)	0	1 (0.35%)	
* Leiomyosarcoma*	1 (0.48%)	0	1 (0.35%)	
* Adenosarcoma*	1 (0.48%)	0	1(0.35%)	
* Mixed*	18 (8.73%)	3 (3.94%)	21 (7.44%)	
* Others*	5 (2.42%)	3 (3.94%)	8 (2.83%)	
**Grade**				
* Grade 1*	44 (21.35%)	27 (35.52%)	71 (25.17%)	0.003
* Grade 2*	53 (25.72%)	26 (34.21%)	79 (28.01%)	
* Grade 3*	80 (33.83%)	13 (17.10%)	93 (32.97%)	
* Others*	26 (12.62%)	8 (10.52%)	34 (12.05%)	
**Ascites or washing cytology**				
* Not done*	48 (24.30%)	10 (13.15%)	58 (20.56%)	0.390
* Negative malignant cells*	114 (55.33%)	44 (57.89%)	158 (56.02%)	
* Positive atypical cells*	15 (7.28%)	7 (9.21%)	22 (7.80%)	
* Positive malignant cells*	29 (14.07%)	14 (18.42%)	43 (15.24%)	
**Oophorectomy**				
* Not done*	10 (4.85%)	6 (7.89%)	16 (5.67%)	0.111
* Unilateral*	2 (0.97%)	3 (3.90%)	5 (1.77%)	
* Unilateral with wedge resection*	6 (2.90%)	0	6 (2.12%)	
* on contralateral side*				
* Bilateral*	187 (90.77%)	66 (86.84%)	253 (89.17%)	
**Pelvic lymphadenectomy**				
* Not done*	35 (16.99%)	5 (6.57%)	40 (14.18%)	0.076
* Unilateral*	4 (1.94%)	0	4 (1.41%)	
* Bilateral*	166 (80.58%)	71 (93.42%)	237(84.04%)	
**Paraaortic lymphadenectomy**				
* Not done*	120 (58.25%)	40 (52.63%)	160 56.73%)	0.124
* Sampling only*	6 (2.91%)	1 (1.31%)	7 (2.48%)	
* Infra-IMA* ^†^	58 (28.15%)	31 (40.78%)	89 (31.56%)	
* Infra-renal*	22 (10.67%)	4 (5.26%)	26 (9.21%)	
**Myometrial invasion**				
* No invasion*	15 (7.28%)	8 (10.52%)	23(8.15%)	0.046
* Superficial invasion*	12 (5.82%)	6 (7.89%)	18 (6.38%)	
* Inner half invasion*	60 (29.12%)	23 (30.26%)	83 (29.43%)	
* Outer half invasion*	78 (37.86%)	32 (42.10%)	110 (39.00%)	
* Full invasion*	41 (19.9%)	7 (9.21%)	48 (17.02%)	
**LVSI^†^**				
* No*	102 (49.51%)	49 (64.47%)	148 (52.48%)	0.100
* Yes*	104 (50.48%)	30 (39.47%)	134 (47.51%)	
**Adnexal metastasis**				
* No*	153 (74.27%)	69 (90.78%)	222 (78.72%)	0.003
* Yes*	53 (25.72%)	7 (9.21%)	60 (21.27%)	
**Intraperitoneal tumor**				
* No*	147 (71.35%)	68 (89.47%)	215 (76.24%)	0.002
* Yes*	59 (28.64%)	8 (10.52%)	67 (23.75%)	
**Pelvic lymph nodes**				
* Right*				
* Yield*	6.7 ± 5.5	7.3 ± 5.2	6.8 ± 5.4	0.365
*Positive for metastasis*	0.7 ± 1.9	0.4 ± 2.0	0.7 ± 2.0	0.241
* Left*				
* Yield*	5.9 ± 4.8	6.4 ± 4.0	6.0 ± 4.6	0.448
* Positive for metastasis*	0.5 ± 1.4	0.3 ± 1.3	0.5 ± 1.4	0.309
**Paraaortic lymph nodes**				
* Yield*	4.4 ± 7.1	4.6 ± 6.2	4.5 ± 6.9	0.808
* Positive for metastasis*	0.8 ± 3.7	0.5 ± 2.8	0.8 ± 3.5	0.462
**Tumor size (cm)**	6.4 ± 4.4	3.9 ± 2.2	5.7 ± 4.1	< 0.001
**Residual tumor**				
* No*	192 (93.02%)	76 (100%)	268 (95.03%)	0.116
* Yes*	10 (4.85%)	0	10 (3.54%)	
**Estimated blood loss (mL)**	478.3 ± 611.7	228.7 ± 189.4	412.3 ± 544.5	< 0.001
**Post-operative care**				
* PACU* **^†^**	202 (98.05%)	76 (100%)	277 (98.22%)	0.224
* ICU* **^†^**	4 (1.94%)	0	4(1.41%)	
**Post-operative RT^†^**				
* Not done*	74 (35.92%)	21 (27.63%)	95 (33.68%)	0.302
* Brachytherapy*	25 (12.13%)	8 (10.52%)	33 (11.70%)	
* Whole pelvic RT* ^†^	96 (46.60%)	38 (50.00%)	134 (47.51%)	
* Paraaortic RT* ^†^	3 (1.45%)	1 (1.31%)	4 (1.41%)	
* Done at other institutions*	4 (1.94%)	5 (6.57%)	9 (3.19%)	
* No data*	4 (1.94%)	3 (3.94%)	7 (2.48%)	

**^†^**MIS, minimally-invasive surgery; RT, radiotherapy; CCRT, concurrent chemoradiotherapy; CT, chemotherapy; FIGO, International Federation of Gynecology and Obstetrics; MMMT, malignant mixed Müllerian tumor; EST, endometrial sinus tumor; IMA, inferior mesenteric artery; LVSI: lymph-vascular space invasion; PACU, post-anesthesia care unit; ICU, intensive care unit.

**Table 3:** Disease free survival, Cox model.

	Hazard ratio	95% confidence interval	*p*-value
**Age at diagnosis (years)**	1.024	1.003 – 1.046	0.027
**Pre-operative CA-125**	0.999	0.999 – 1.000	0.132
**Types of hysterectomy**			
* Type I*	1		
* Type II*	1.758	0.994 – 3.107	0.052
* Type III*	0.953	0.430 – 2.112	0.905
**FIGO^†^ stage**			
* Stages 1-2*	1		
* Stages 3-4*	2.228	1.228 – 4.040	0.008
**Grade**			
* Grade 1*	1		
* Grade 2-3*	2.646	1.260 – 5.539	0.010
**Tumor size**	1.070	1.015 – 1.128	0.011
**Lymph-vascular space invasion**	1.705	1.056 – 2.753	0.029
**Types of surgery**			
* Laparotomy*	1		
* Minimally-invasive surgery*	0.696	0.371 – 1.306	0.260
**Types of colpotomy**			
* Intracorporeal*	1		
* Transvaginal*	0.317	0.058 – 1.722	0.183

^†^FIGO, International Federation of Gynecology and Obstetrics.

**Table 4:** Overall survival, Cox model.

	Hazard ratio	95% confidence interval	*p*-value
**Age at diagnosis (years)**	1.038	1.013 – 1.064	0.002
**Pre-operative CA-125**	1.000	0.999 – 1.001	0.750
**Types of hysterectomy**			
* Type I*	1		
* Type II*	2.010	1.041 – 3.883	0.038
* Type III*	1.448	0.611 – 3.621	0.381
**FIGO^†^ stage**			
* Stages 1-2*	1		
* Stages 3-4*	1.777	1.002 – 3.152	0.047
**Grade**			
* Grade 1*	1		
* Grade 2-3*	2.491	1.383 – 4.485	0.030
**Tumor size**	1.048	0.966 – 1.136	0.259
**Lymph-vascular space invasion**	2.512	1.358 – 4.645	0.003
**Types of surgery**			
* Laparotomy*	1		
* Minimally-invasive surgery*	1.661	0.890 – 3.100	0.111
**Types of colpotomy**			
* Intracorporeal*	1		
* Transvaginal*	1.241	0.174 – 8.878	0.830

^†^FIGO, International Federation of Gynecology and Obstetrics.

Among the patients in the MIS group, 45 patients underwent surgery in the form of LAVH (including both LAVH and LARVH) while the rest received surgery in the form of TLH (including TLH, LRH, and robotic hysterectomy). The main difference between the two types of surgical approach was how to ligate the uterine arteries and perform colpotomy. Subgroup analysis was performed to rule out the possibilities that each surgical method balances advantages or disadvantages of one another. Perioperative outcomes did not reveal significant differences between the two groups (**Supplementary Table 1**) while the Cox proportional hazards model showed that the differences of surgical approach did not affect survival outcomes as seen in **Tables 3** and **4**. The two groups showed significantly different perioperative outcomes (**Table 5**). While the total operation time was significantly longer in the laparotomy group compared to the MIS group, the MIS group demonstrated less blood loss during the operations, lower rates of transfusion during or after surgery, less post-operative pain measured by NRS, and shorter duration of hospital stay. Peri-operative complications did not differ between the two groups. We experienced 4 distal ureteral injuries and two bladder serosal injuries that were all found intraoperatively and repaired. One patient in the laparotomy group had vaginal vault bleeding on her post-operative day 1, which was managed by gauze compression. One patient from each group had vaginal vault dehiscence, which required re-suture. All post-operative bleeding patients received red blood cell transfusion in addition to tranexamic acid infusion but none required re-operation. Abdominal wound complications were found in three patients which included infection and dehiscence (at the level of subcutaneous tissue with intact fascia). However, it was apparent that the longer operation time of the laparotomy group was due to the advanced stages of the patients who required more surgical procedures with high complexity. Four patients in the laparotomy group went to the intensive care unit (ICU) post-operatively. The length of stay in the ICU of all patients was less than 24 hours and the main reason for the stay was for close surveillance. In the present institution, anesthesiologists often recommend post-operative ICU care for patients who underwent extensive surgical procedures even if their vital signs and hematologic parameters stay stable, which was the case for all 4 patients in the present study. The mean estimated blood loss during the operations of them was 387 mL.

**Table 5:** Perioperative outcomes.

	Laparotomy	MIS^†^	*p*-value
**Intraoperative factors**			
* Anesthesia time (min)*	198 (80 – 383)	259 (128 – 724)	0.029
* Operation time (min)*	460 (65 – 321)	222 (93 – 623)	0.008
**Blood transfusion required**			
* RBC* **^†^** *transfusion during or after surgery*	42 (20.39%)	4 (5.26%)	0.002
* Hemoglobin drop* ^††^ *on POD* ^†^ *#1*	1.8 (-0.2 – 4.2)	1.25 (-0.2 – 3.5)	0.319
**Postanesthesia care unit (PACU)**			
* PACU stay (min)*	90 (50 – 190)	80 (48 – 130)	0.133
**Perioperative complications**			
* Distal ureteral injury*	3	1	0.935
* Bladder injury*	2	0	
* Vaginal vault bleeding*	1	0	
* Vaginal vault dehiscence*	1	1	
* Postoperative bleeding*	4	2	
* Abdominal wound complications*	2	1	
**Postoperative floor numeric rating score (NRS)**			
* NRS 0 – 6 hours after surgery*	5 (2 – 8)	3 (2 – 8)	0.054
* NRS 12 – 24 hours after surgery*	3 (2 – 6)	3 (2 – 5)	0.019
**Hospital stay (days)**	7.1 ± 4.7	4.4 ± 2.3	0.002

^†^MIS, Minimally-invasive surgery; RBC, red blood cell; POD, postoperative day.

^††^Defined as postoperative hemoglobin levels subtracted from preoperative hemoglobin levels.

## Discussion

In the present study, we evaluated the patients with endometrial cancer that involved the cervix on final pathology and compared the outcomes between those who received MIS *vs*. laparotomy. It was found that MIS was not associated with decreased survival outcomes. Furthermore, perioperative outcomes mainly favored MIS over laparotomy demonstrating the benefits of MIS that were also seen in numerous previous studies (10–12).

Previous studies in the literature have consistently demonstrated the non-inferiority of MIS in terms of oncologic outcomes in endometrial cancer compared to laparotomy. Among the studies, the LAP2 study by the Gynecologic Oncology Group is the largest randomized controlled trial, in which the authors compared MIS *vs*. laparotomy in 2,616 patients (7). In that study, patients with clinical stages I to IIA were randomly allocated to laparoscopy versus laparotomy. The trial demonstrated the feasibility and safety of MIS by showing almost identical 5-year overall survival at 89.8%. Other oncologic outcomes were also comparable between the two groups. Among the patients included in the study, 99 patients were found to have FIGO stage II on final pathologic evaluations (65 patients in the MIS group *vs*. 34 patients in the laparotomy group). Subgroup analysis of those patients also demonstrated no decrement of survival in the MIS group.

Another landmark randomized controlled trial evaluated 760 women with FIGO stage I endometrioid endometrial cancer (6). The results of the trial also supported the use of laparoscopic hysterectomy by showing equivalent DFS at 4.5 years and no difference in OS. Among the patients included, 72 patients were found to be FIGO stage II on final pathologic evaluation (32 patients in the MIS group *vs*. 45 patients in the laparotomy group) and there was no statistically significant difference between the MIS group *vs*. the laparotomy groups in any of subgroup analysis including FIGO stages.

Endometrial cancer is commonly confined to the uterus at diagnosis. According to the data from the National Cancer Institute’s Surveillance, Epidemiology, and End Results (SEER) program, FIGO stage I disease was found in 73% of patients, and 10% had stage II disease among all endometrial cancer patients (13). The 26^th^ Annual Report of the FIGO on 9,386 endometrial cancer patients also demonstrated that 83% of patients were stage I – II (14). Cervical involvement of endometrial cancer is often not detected prior to hysterectomy and superficial involvement of the cervix by tumor may not be diagnosed by frozen section analysis. Only about 40% of the patients in the present study showed the cervical involvement of tumor on pre-operative imaging. Therefore a significant portion of the patients who were initially thought to have FIGO stage I disease before surgical treatment are eventually diagnosed with FIGO stage II after final pathology evaluation. This may generate aforementioned concerns for both surgeons and patients. However, the results from the present study, combined with previous findings from the literature, reassures that laparoscopic surgery can safely be performed for the patients whose tumor invades the uterine cervix. Furthermore, recent studies demonstrated that laparoscopic surgery did not impair OS in more advanced stages of endometrial cancer such as stage IIIC suggesting that the indication to MIS might be broadened to more advanced disease status, provided that the entire disease is removed (15). In other words, data are being accumulated supporting the use of MIS in endometrial cancer. Studies to date evaluating a variety of factors such as histology, grade, stage, and nodal status, did not reveal any evidence of a particular subgroup of patients that should not be treated with laparoscopy. Moreover, recent studies in robotic surgery revealed that elderly patients in particular may benefit the advantages and favorable perioperative outcomes of MIS when multidisciplinary approach is taken to provide the best management pathway (16, 17).

One of the potential explanations for the decreased survival outcomes seen in the patients who were treated laparoscopically for early cervical cancer in the LACC trial is the use of uterine manipulator, which might increase the propensity for tumor spillage. During the study period, the surgeons at the present institution also used uterine manipulators routinely. However, the device was installed only after the electrocoagulation of the isthmus of the fallopian tubes with bipolar forceps. The colpotomy was performed by either intracorporeal approach or transvaginal approach at the surgeons’ discretion. The surgeons were not particularly concerned in regards to the increased likelihood of tumor recurrence in patients whose tumor invaded the cervix. The report of the LACC trial called into question whether the decreased survival of MIS would apply to endometrial cancer. The results of the present study showed the methods of colpotomy were not associated with survival outcomes. Further investigation is warranted to explain the difference in the observations of the adverse effects of uterine manipulators in the two different types of malignancies.

The present study adds valuable information to the literature in that it is the first study to compare MIS *vs*. laparotomy in patients with endometrial cancer whose tumor involves the uterine cervix. It showed comparable survival outcomes between the two groups. It also has limitations. The number of patients evaluated in the present study is still relatively small to generalize the results to all stages of endometrial cancer patients. Another limitation of the study is that, due to the retrospective design, there might have been selection bias of the patients. It is evident that the patients with advanced stages of endometrial cancer were more likely to receive laparotomy. This could not exclude that patients with more advanced FIGO stages, higher histology grades, deeper myometrial invasion, adnexal and intraperitoneal metastases, and larger tumor size underwent laparotomy, which makes them not comparable to those treated by MIS. However, already given the positive evidence of MIS from previous studies, it was ethically not feasible to randomize the patients into MIS *vs.* laparotomy. Therefore, it was our best effort to analyze this issue retrospectively with collected data from our patients. In order to minimize the potential bias, we performed the Cox proportional hazards model with other variables that are already known to affect patient survivals in endometrial cancer. Although statistical methods were implemented to control this factor, this certainly limits the interpretation of the results and remains as the main limitation of the study.

Despite the presence of the aforementioned limitations, the results of the present study along with those from other previous studies suggest that surgical staging can be performed laparoscopically in patients with endometrial cancer that involves the cervix of the uterus. Long-term survival analysis should be supported by randomized controlled studies to demonstrate that laparoscopic approach may be an acceptable alternative to laparotomy in this patient group.

## Data Availability Statement

The raw data supporting the conclusions of this article will be made available by the authors, without undue reservation.

## Ethics Statement

The studies involving human participants were reviewed and approved by Samsung Medical Center. Written informed consent for participation was not required for this study in accordance with the national legislation and the institutional requirements.

## Author Contributions

The present study was designed, directed and coordinated by Y-YL, as the principal investigator. Y-YL provided conceptual and technical guidance for all aspects of the project. JJ and JN planned and performed the analyses of the data with CC, T-JK, and J-WL. The data were collected by CC, T-JK, J-WL, B-GK, and D-SB. The manuscript was written by JJ and JN and commented on by all authors. All the authors meet the recommendations for the conduct, reporting, editing, and publication of scholarly work in medical journals provided by the International Committee of Medical Journal Editors. All authors contributed to the article and approved the submitted version.

## Funding

This work was supported by the National Research Foundation of Korea (NRF) grant funded by the Korean government (MSIT) (2019R1F1A1063567).

## Conflict of Interest

The authors declare that the research was conducted in the absence of any commercial or financial relationships that could be construed as a potential conflict of interest.
