# Supplementary material for: Minimally-Invasive Versus Abdominal Hysterectomy for Endometrial Carcinoma With Glandular or Stromal Invasion of Cervix
Source: Front Oncol. 2021 May 20;11:670214. doi: 10.3389/fonc.2021.670214 (PMC8173145; doi:10.3389/fonc.2021.670214)
Supplement: Supplementary file 1 [file DataSheet_1.docx]

**Supplementary table 1. Disease free survival, Cox model (stage II patients only)**

| **Multivariate analysis** | **Hazard ratio** | **95% confidence interval** | ***p*-value** |
| --- | --- | --- | --- |
| **Age (years)** | 0.982 | 0.894 – 1.079 | .707 |
| **Types of hysterectomy**  *Type I*  *Type II*  *Type III* | 1  1.736  4.718 | 0.143 – 21.049  0.075 – 24.407 | .665  .112 |
| **Preoperative CA-125 (U/mL)** | 0.990 | 0.977 – 1.003 | .137 |
| **Histological grade**  *Grade 1*  *Grade 2*  *Grade 3* | 1  0.081  0.368 | 0.004 – 1.847  0.031 – 4.360 | .115  .428 |
| **Cervical involvement**  *Glandular*  *Stromal* | 1  0.378 | 0.038 – 3.578 | .406 |
| **Tumor size (cm)** | 1.271 | 0.093 – 1.788 | .169 |
| **Myometrial invasion**  *Inner half*  *Outer half* | 267.991  1156.938 | 0 – NA  0 – NA | .966  .967 |
| **Lymph-vascular space invasion** | 5.188 | 0.476 – 56.504 | .177 |
| **Minimally-invasive surgery *vs*. laparotomy** | 0.172 | 0.016 – 1.870 | .148 |

**Supplementary table 2. Overall survival, Cox model (stage II patients only)**

| **Multivariate analysis** | **Hazard ratio** | **95% confidence interval** | ***p*-value** |
| --- | --- | --- | --- |
| **Age (years)** | 1.034 | 0.871 – 1.227 | .701 |
| **Types of hysterectomy**  *Type I*  *Type II*  *Type III* | 1  1.939  31.403 | 0.080 – 46.920  1.345 – 733.214 | .684  .032 |
| **Preoperative CA-125 (U/mL)** | 0.989 | 0.972 – 1.006 | .191 |
| **Histological grade**  *Grade 1*  *Grade 2*  *Grade 3* | 1  0  1.119 | 0 – NA  0.018 – 70.686 | .691  .958 |
| **Cervical involvement**  *Glandular*  *Stromal* | 1  3.822 | 0.077 – 190.883 | .502 |
| **Tumor size (cm)** | 0.869 | 0.532 – 1.428 | .573 |
| **Myometrial invasion**  *Inner half*  *Outer half* | 81.628  89.415 | 0 – NA  0 - NA | .982  .982 |
| **Lymph-vascular space invasion** | 345.795 | 3.528 – 33892-140 | .012 |
| **Minimally-invasive surgery *vs*. laparotomy** | 5.115 | 0.241 – 108.543 | .295 |

**Supplementary table 3.** Perioperative outcomes between LAVH**^†^** *vs*. TLH**^†^**

|  | **LAVH^†^/LARVH^†^**  **(N = 45)** | **TLH^†^/LRH^†^/Robot surgery**  **(N = 31)** | ***p*-value** |
| --- | --- | --- | --- |
| **Intraoperative factors**  *Anesthesia time (min)*  *Operation time (min)* | 286 (128 – 582)  254 (93 – 321) | 241 (131 – 724)  220 (127 – 623) | 0.422  0.108 |
| **Blood transfusion required**  *RBC***^†^** *transfusion during or after surgery*  *Hemoglobin drop***^††^** *on POD***^†^** *#1* | 2 (4.44%)  1.1 (-0.2 – 3.3) | 2 (6.45%)  1.1 (-0.1 – 3.5) | 0.700  0.263 |
| **Postanesthesia care unit (PACU)**  *PACU stay (min)* | 75 (48 – 130) | 83 (52 – 111) | 0.531 |
| **Perioperative complications**  *Distal ureteral injury*  *Bladder injury*  *Vaginal vault bleeding*  *Vaginal vault dehiscence*  *Postoperative bleeding*  *Abdominal wound complications* | 0  0  0  0  1  0 | 1  0  0  1  1  1 | 0.065 |
| **Postoperative floor numeric rating score (NRS)**  *NRS 0 – 6 hours after surgery*  *NRS 12 – 24 hours after surgery* | 3 (2 – 8)  3 (2 – 5) | 3 (3 – 7)  3 (2 – 5) | 0.157  0.214 |
| **Hospital stay (days)** | 3.9 ± 2.7 | 4.0 ± 2.1 | 0.857 |

**^†^**LAVH: laparoscopy-assisted vaginal hysterectomy, LARVH: laparoscopy-assisted radical vaginal hysterectomy, TLH: total laparoscopic hysterectomy, LRH: laparoscopic radical hysterectomy, RBC: red blood cell, POD: postoperative day. **^††^**Defined as postoperative hemoglobin levels subtracted from preoperative hemoglobin levels.
